# Medical Advertising on the Continent

**Published:** 1899-04-29

**Authors:** 


### Medical Advertising on the Continent.

The *English and American Gazette* is evidently very angry with us for commenting as we have done on the advertising tendencies of the Continental Anglo-American Medical Society. For ourselves we do not pretend to know what is the connection between that newspaper and that society, but it seems to be a fairly intimate one, if one may judge by the kind manner in which the one advertises the other—and all for nothing! We presume, then, that the statements made by our contemporary may be taken as more or less authoritative, and as they are of an interesting nature we would draw attention to one or two of them.

In regard to the functions of the Continental Anglo-American Medical Society, we are told that "It has one single article of association, which runs thus: 'Objects of the Association.—To promote social intercourse and maintain good fellowship between English and American physicians who practise abroad.'"

How this good fellowship works out we are left to gather further on. "Before the Association existed the scattered units throughout Europe . . . . had no means of making themselves known to the public except by going behind their rules of etiquette and paying for an advertisement! They did this in sheer desperation—in many instances." Of course, if medical men advertised in England we should know what to think of them. Abroad, however, the paths of the advertiser appear to be made curiously smooth. "Last winter," says the editor of the *English and American Gazette*, "when a large card containing a list of the members of the association, and which was found (rarely) in the hotels for which it was destined, was discontinued, the business director of this newspaper suggested that the *English and American Gazette* would guarantee a weekly (instead of an intermittent) appearance of the entire list, on the same payment being made (for space and composition) that was expended in the society's cards. But the writer—on being told by the honorary secretary that the majority of the members considered such payment as not etiquette—replied: 'Though we sacrifice a large amount of valuable space weekly, yet we will continue so to do, for we consider the public gain of far more import than what we may lose.'" Really, this is too charming! It makes us long to live abroad where we can be advertised all for nothing by these complaisant business directors, although we must confess to a little surprise that, when doing so much for "the public gain," this newspaper did not go so far as to publish the names of all the medical men practising in the towns mentioned, instead of just advertising the members of the society. Speaking seriously, however, we have here the statement that this society did as a fact send "a large card containing the list of members of the association" to the various hotels, and further on we have the admission, in regard to the list published week by week in the *English and American Gazette*, that the Royal College of Physicians would certainly put a veto on the Anglo-American Medical Society "if such list was paid for at the advertisement rate." We quite accept the statement of the editor that although, in conjunction with his paper, he has since 1897 published the list of "all those belonging to the association practising throughout Europe," he has "neither asked for nor received the slightest monetary consideration." But, while expressing our wonder at Continental methods of newspaper management which will allow of such an arrangement, we may say that we do not think the absence of monetary consideration makes very much difference to the ethics of advertising. We cannot imagine that it is right to do through a friend what it is wrong to do through an agent. In fact we think, as we said, that the society "ought to be able to protect itself from such a misuse of its list of members." Perhaps, however, after all, it is not so easy to control the vagaries of a foreign journal as we may have imagined, for in this very article in the *English and American Gazette*, April 8th, 1899, we find the following statement in regard to the Continental Anglo-American Medical Society: "The last honorary English presidents of the society were—Sir S. Wells, Bart., F.R.S.; Sir Joseph Lister, Bart., P.R.S.; Sir Richard Quain, Bart., P.R.S.; Sir Samuel Wilks, F.R.S."

We cannot congratulate our contemporary on its accuracy. We think we have seen a later list than that. But, in any case, what does the publication of this list mean? Are Lord Lister and Sir Samuel Wilks still honorary presidents of this much-advertising society? If this is so, the matter is even more serious than we had thought. Or does the editor hold firm by the little word "were," and is this merely an attempt to throw dust in the eyes of the public, and to make them believe that all these things are done under the ægis of great names?

